# Retraction Note: Computational simulation and modelling of uranium extraction using tributylphosphate through membrane extractor

**DOI:** 10.1038/s41598-026-56027-1

**Published:** 2026-06-03

**Authors:** Rahmad Syah, Dadan Ramadan, Marischa Elveny, Yan Cao, Afrasyab Khan, Hamid Abdi, Mahdi Ghadiri

**Affiliations:** 1 Faculty of Engineering, Universitas Medan Area, Medan, Indonesia; 2 Data Science & Computational Intelligence Research Group, Universitas Sumatera Utara, Medan, Indonesia; 3 School of Mechatronic Engineering, Xi’an Technological University, Xi’an, 710021 China; 4 Institute of Engineering and Technology, Department of Hydraulics and Hydraulic and Pneumatic Systems, South Ural State University, Lenin Prospect 76, Chelyabinsk, 454080 Russian Federation; 5 Department of Chemical Engineering, Petroleum University of Technology, Ahwaz, Iran; 6 Department of Chemical Sciences, Bernal Institute, University of Limerick, Limerick, Ireland

Retraction of: *Scientific Reports* 10.1038/s41598-021-97379-0, published online 08 September 2021

The Editors have retracted this Article.

After publication, concerns were raised about some of the references not being relevant to the context in which they are cited. Additional investigation by the Editors revealed concerns around the authorship of this Article. The Authors did not provide a satisfactory explanation to these concerns. The Editors have therefore lost confidence in this Article.

Mahdi Ghadiri did not explicitly state if they agree or disagree with this retraction. Rahmad Syah, Dadan Ramadan, Marischa Elveny, Yan Cao, Afrasyab Khan, and Hamid Abdi did not reply to the Editors’ correspondence about the retraction.

